# Comparing learners’ knowledge, behaviors, and attitudes between two instructional modes of computer programming in secondary education

**DOI:** 10.1186/s40594-021-00311-1

**Published:** 2021-09-23

**Authors:** Dan Sun, Fan Ouyang, Yan Li, Caifeng Zhu

**Affiliations:** 1 College of Education, Zhejiang University, #866, Yuhangtang Rd., Hangzhou, 310058 Zhejiang China; 2 The Affiliated School of the College of Education, Zhejiang University, #118, Fanghua Rd., Hangzhou, 310053 Zhejiang China

**Keywords:** STEM education, Unplugged programming, Process-oriented analysis, Behavioral pattern analysis, Secondary education

## Abstract

**Background:**

Unplugged programming is proved to be an effective means to foster the learner-centered programming learning. In addition to the final tests, learners’ programming knowledge, skills, and capacities are primarily demonstrated throughout the programming process, particularly in the situation when they encounter challenges and problems. However, few studies examine how learners engage in the programming processes and to what extent unplugged programming fosters learning. This research used a quasi-experimental design to investigate two instructional modes in China’s secondary education, namely, the instructor-directed lecturing and the learner-centered unplugged programming. Based on an analytical framework, this research used mixed methods to compare learners’ knowledge, behaviors, and attitudes under these two instructional modes.

**Results:**

The research results revealed discrepancies between two instructional modes. First, learners in the unplugged programming class achieved significantly higher scores on the programming knowledge assessment, compared to learners in the traditional lecturing class. Second, compared to the traditional lecturing class, learners in the unplugged programming class had higher test scores of the computational thinking skills, particularly on the cooperativity dimension. Next, discrepancies of in-class behaviors showed that learners in the unplugged programming class had frequent behaviors of listening to the instructor’s instructions and discussing with peers, while learners in the instructor-directed class had frequent behaviors of listening to instructor, taking notes, and irrelevant activities. Learners’ self-reported attitudes in the unplugged programming indicated a higher level of confidence than learners in the traditional lecturing class. Overall, this research revealed that the learner-centered unplugged programming had potential to improve learners’ programming knowledge, behaviors, and attitudes compared to the traditional instructor-directed lecturing of programming.

**Conclusions:**

As a feasible and easy-to-use instructional activity in computer science education, unplugged programming is encouraged to be integrated in formal education to increase learners’ programming interests, motivations, and qualities. This quasi-experimental research compared learners’ programming knowledge, behaviors, and attitudes under two instructional modes. The results revealed critical discrepancies between two instructional modes on learners’ knowledge gains, in-class behaviors, and changes of attitudes towards programming. Pedagogical and analytical implications were provided for future instructional design and learning analytics of computer programming education.

**Supplementary Information:**

The online version contains supplementary material available at 10.1186/s40594-021-00311-1.

## Introduction

As one strand of the science, technology, engineering and mathematics (STEM) education, computer programming has positive influences on advancing learners’ computational thinking (CT) skills (Sun et al., 2021a, b), fostering their motivation and engagement (Schnittka et al., 2015), and improving their computer science career interests (Chittum et al., 2017). In formal education, the instructor-directed lecturing is a widely used instructional mode, through which instructors transmit computer programming knowledge to learners with oral presentations (Wu & Wang, 2017). Although this instructional approach helps learners gain computer programming knowledge, instructors encounter many challenges during actual programming practices, such as how to decrease learners’ frustration and failure, how to sustain their programming interests and motivations, and how to eventually improve their programming skills and capacities (Falloon, 2016; Looi et al., 2018; Tom, 2015). To address those challenges, emerging instructional strategies, e.g., unplugged, game-based, or project-based programming, have been used in informal learning to transform the instructor-directed lecturing of programming knowledge to the pragmatic, learner-centered programming practices (Brackmann et al., 2017; Hosseini et al., 2019; Nurbekova et al., 2020).

Among those practical strategies, unplugged programming is a hands-on programming activity without the supports of computers or other electronic technologies to contextualize computational concepts and algorithms through physical or kinesthetic activities (e.g., Alamer et al., 2015; Gouws et al., 2013; Thies & Vahrenhold, 2013). Research argues that unplugged programming activities can simplify computational concepts for learners and, therefore, promote their programming engagement, motivation, and interest (Alamer et al., 2015; Looi et al., 2018). During the programming process, learners usually encounter programming challenges and problems; to solve programming problems, they need to pose and answer questions, share and construct knowledge, and create programming solutions or products through individual learning or peer interaction (Lewis, 2012; Sun et al., 2021a, b; Wu et al., 2019). However, few studies actually examine how learners engage in the programming practices from a process-oriented perspective, and to what extent unplugged programming activities foster learner learning (del Olmo-Muñoz et al., 2020; Grover et al., 2019; Huang & Looi, 2020). The process-oriented perspective focuses on details of how students coordinate their communications, discourses, and behaviors during actual instruction and learning processes (Pereira et al., 2020; Sun et al., 2021a, b; Wu et al., 2019). Correspondingly, the process-oriented analysis stresses the micro-level, fine-grained analysis of students’ behavioral, cognitive, metacognitive activities during the programming practice, which is beneficial for researchers to gain a holistic insight into how programming activities progress.

In response to this research gap, this research used a quasi-experimental research supported with mixed methods to implement and investigate the learner-centered unplugged programming in China’s secondary education and compared the effects of the unplugged programming activities on learner’s learning with the traditional instructor-directed lecturing mode. Specifically, mixed methods (i.e., video analysis, lag sequential analysis, statistical analysis, and thematic analysis) were used to analyze and compare learners’ gains of programming knowledge, in-class behaviors, and attitudes towards programming between two instructional modes. Based on empirical results, this research proposed a holistic insight of pedagogical and analytical implications for computer programming education.

## Literature review

Grounded upon the constructivist perspective, learning is an active, constructive process, through which learners actively construct their own understandings through interacting with peers, resources, and technologies (Papert, 1991). Contextualization of sophisticated computational algorithms is a means to alleviate the difficulties of learners’ conceptual understandings, and, therefore, stimulate learners’ active learning and construction of programming knowledge (Bransford et al., 2000; Falloon, 2016). A major approach to contextualize programming is the unplugged programming that exposes learners to computational concepts and algorithms without the support of computers (Bell et al., 2009). In the hands-on, unplugged activities, learners conceptually engage in understanding relevant programming knowledge through a series of contextualized materials (e.g., logic games, cards, strings, or physical actions). The unplugged programming is mostly used in the informal learning contexts to engage novice learners in computer programming (Taub et al., 2012; Thies & Vahrenhold, 2013). During the programming process, learners’ programming knowledge, skills, and capacities can be demonstrated, particularly in the situation when they encounter challenges and problems that require deliberate problem-solving and meaning-making process (Wu et al., 2019). In K-12 formal educational context, the main instructional approach of computer programming is still the instructor-directed lecturing, sometimes followed with learners’ programming practices on computers (Panwong & Kemavuthanon, 2014).

Empirical research has indicated that, compared to the traditional instructor-directed lecturing, unplugged programming has potential to foster learners’ programming knowledge, active engagement, and positive attitudes. First, unplugged programming can improve learners’ computational thinking (CT) skills and programming knowledge acquisitions. For example, Alamer et al. (2015) reported that unplugged activities succeeded in simplifying key programming concepts to shape and deepen learners’ understandings of the programming knowledge. Ballard and Haroldson (2021) also summarized the effects of using non-programming, unplugged approaches to teach programming skills and concepts (e.g., abstraction, generalization, decomposition, algorithmic thinking, debugging). Second, research finds that unplugged programming has potential to improve learners’ active engagement. For example, through video analysis, Looi et al. (2018) found that unplugged activities helped all group learners engage in the explorations of the sorting algorithms, which resulted in good programming performances on this algorithm. Tsarava et al. (2018) designed board games to increase children’s motivation for programming learning, and found that unplugged activities helped keep children engaged in the programming game. Third, a series of studies have been conducted to examine the benefits of unplugged programming for promoting learners’ positive attitudes. For example, Hermans and Avvaloglou (2017) found that, compared with learning in Scratch, learners who started with unplugged lessons were more confident of their capacities to understand the programming concepts. Mano et al. (2010) designed in-class unplugged programming activities and found an improvement in the interest levels in computing among learners. Overall, unplugged programming activities have potential to promote learners’ programming knowledge, programming engagement, and positive attitudes towards programming.

Although relevant studies argue that unplugged programming activities can foster learners’ active engagement, few studies actually examine how learners engage in the programming practices from a process-oriented perspective and to what extent unplugged programming activities foster learner’s learning. Most studies focus on summative assessment of learners’ programming knowledge acquisitions, improvements of CT skills, or self-reported perceptions towards programming. For example, Brackmann et al. (2017) designed a quasi-experiment research to examine the effectiveness of unplugged activities on the development of CT skills in primary schools, and found learners who took part in the unplugged activities enhanced their CT skills significantly, compared to their peers in control groups. Gardeli and Vosinakis (2017) continuously observed learners’ individual and group behaviors during unplugged visual programming, and reported that the unplugged activity was an engaging and collaborative approach to improve learners’ satisfaction and enjoyment. Torres-Torres et al. (2019) used instructor’s informal observations and found the unplugged programming class managed to build routes of algorithm learning and achieve a high level of complexity in the codes. Saxena et al. (2020) used field notes to record learner performances and interactions as well as instructor’s instructional practices during unplugged and plugged activities, to provide learners with concrete guidance and support for subsequent programming activity. Taken together, most of those studies use the summative assessments or informal observations to examine learners’ programming skills and engagement levels, but do not examine learners’ engagement during the programming practices from a process-oriented perspective. Since programming requires a deliberate problem-solving, meaning-making, and knowledge-construction process (Lewis, 2012; Sun et al., 2021a, b; Wu et al., 2019), it is beneficial to empirically examine how unplugged programming influences learners’ programming from the process-oriented perspectives, to provide a holistic picture of learners’ programming behaviors, communications, and interactions (Sun et al., 2021a, b; Wu et al., 2019).

Multiple analytical methods have been utilized in previous empirical research to analyze and demonstrate varied aspects of the programming processes. Berland et al. (2013) used learning analytics and data mining to examine details of how learners progressed from exploration, tinkering, to refinement during the learning processes. Results showed that learners in the exploration period produced more low quality programs, while the other two periods had much higher level of quality program states. Wu et al. (2019) used a quantitative ethnography approach to analyze the collaborative programming between a high‐performing and a low‐performing team. Results indicated that the high‐performing team exhibited the systematic CT skills, whereas the low‐performing team’s CT skills were characterized by tinkering or guessing. Sun et al., (2021a, b) used mixed methods (e.g., click stream analysis, lag-sequential analysis, quantitative content analysis) to analyze three contrasting pairs’ collaborative programming behaviors, discourses, and perceptions. Results characterized the high-, medium-, and low-ranked pairs with different characteristics on the social interactive, cognitive engagement and final performing dimensions. Those studies indicate that multiple methods can be used to conduct the process-oriented analysis of computer programming, which is beneficial to demonstrate varied dimensions of the learning processes. As a complementary, the traditional, summative assessment (e.g., final tests) can help reveal learners’ direct performances of computer programming knowledge or skills. Following the analytical trend, this research uses a mixed method approach to reveal the effectiveness of unplugged programming from the summative and process-oriented perspectives.

To address those research and practice gaps, this quasi-experimental research applied two instructional modes, namely, the instructor-directed lecturing of programming and the learner-centered unplugged programming in China’s secondary education to improve computer programming education quality. Furthermore, this research used mixed methods to analyze and compare the effects of novice learners’ programming in those two instructional modes to inform instructional design of computer programming. The effects of learners’ computer programming were examined from the summative and process-oriented perspectives. Specifically, from the summative perspective, this research investigated learners’ programming knowledge gains and changes of attitudes before and after two instructional modes. From the process-oriented perspectives, this research examined learners’ in-class behaviors during instruction and learning activities under two instructional modes. Mixed methods were used, including statistical analyses of knowledge test and survey data, sequential analysis of in-class video data, and qualitative analysis of interview data. Based on the results, this research proposed pedagogical and analytical implications for future instructional design and research analytics of computer programming.

## Research methodology

### Research purposes and questions

The research purpose was to compare effects of learners’ programming learning between two instructional modes, namely, the instruction with traditional instructor-directed lecturing (IDL) and the instruction with learner-centered unplugged programming (UPP). We compared the difference of learners’ knowledge gains, in-class behaviors, and changes of attitudes between two instructional modes. There were three research questions:

- RQ 1. How did the impact of UPP on learners’ computer programming knowledge and skills differ from the impact of IDL?
- RQ 2. How did the impact of UPP on learners’ learning behaviors differ from the impact of IDL?
- RQ 3. How did the impact of UPP on learners’ positive attitudes towards programming differ from the impact of IDL?

### The research analytical framework

**Fig. 1:**
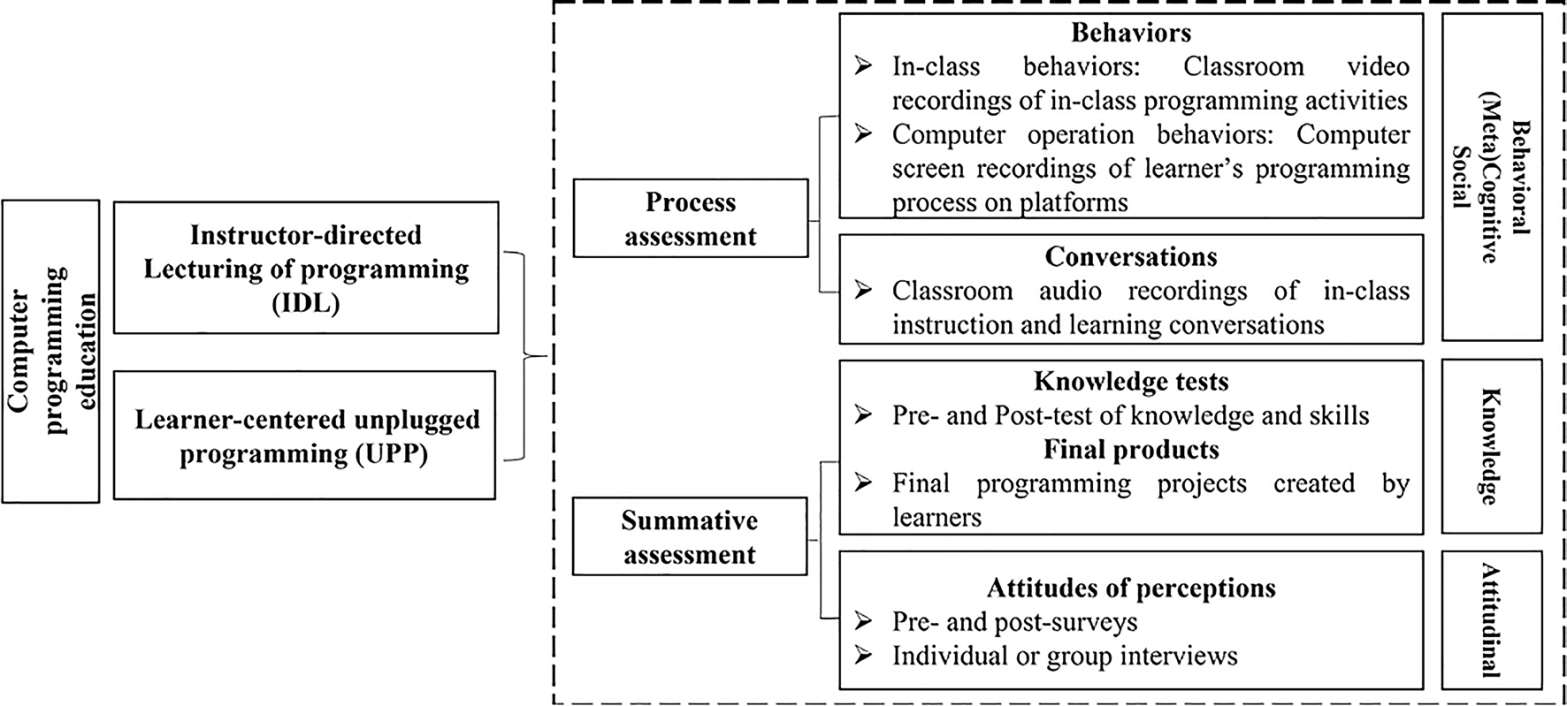
Analytical framework

This research proposed an analytical framework to investigate the differences between instructor-directed lecturing of programming (IDL) and learner-centered unplugged programming (UPP) from the process and summative perspective (see Fig. 1). On the process assessment perspective, research can collect behavioral data, including in-class behaviors (classroom video recordings of in-class programming activities) and computer operation behaviors (computer screen recordings of learner’s programming operations). Classroom video analysis and click-stream analysis can be applied to analyze behavior data, respectively. In addition, classroom audio recordings can capture in-class conversations from learners and the instructor, where quantitative content analysis, lag-sequential analysis and ethnographic interpretations could be applied to examine discourse patterns and characteristics. On the summative assessment perspective, programming knowledge data (e.g., pre- and post-tests) and final products (e.g., programming projects) can be collected as performance data and statistics can be used to examine the significance of performance changes. Moreover, as the Additional file 1, Additional file 2, learner attitudes (including data from pre-, post-surveys or interviews) can be used to further understand learners’ perceptions about programming. Taken together, this analytical framework provides an integration of the process and summative assessments for computer programming education.

### Research context, participants, and instructional procedures

The research context is a compulsory course titled “Creative Programming Algorithms” offered at a junior high school during Spring 2020 in the Eastern area of China. Under the COVID-19 period, learners were not allowed to get access to the computer labs; instead, the classes were offered in a normal classroom with interactive whiteboards. This research used a quasi-experimental design to investigate learners’ knowledge gains, in-class behaviors, and attitudinal changes under the control condition (the instructor-directed lecturing of programming; IDL) and the experimental condition (the learner-centered unplugged programming; UPP).

**Fig. 2:**
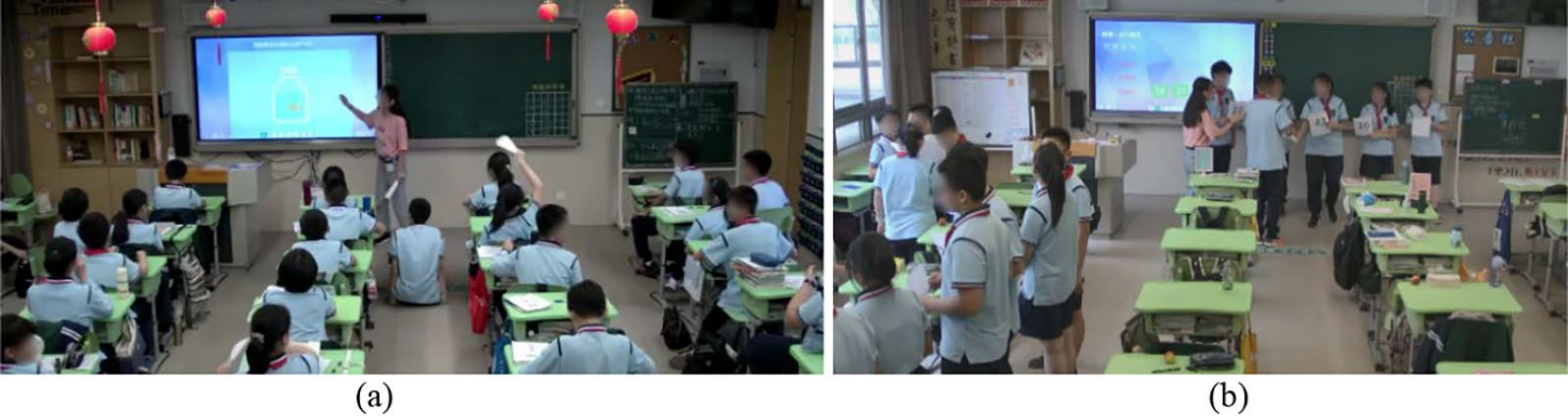
Control class: IDL (**a**) and the experimental class: UPP (**b**)

There were 31 learners (female = 16; male = 15) in the control IDL class and 32 learners (female = 19; male = 13) in the experimental UPP class. Control and experimental classes were randomly assigned; learners in two classes were not informed of the different treatments. Classes were taught by the same instructor (the fourth author), who maintained the same teaching style under two conditions, offered the same instructional materials to learners, and used the same teaching guidance for each class, except the use of the unplugged programming activities in the experiment condition. The instructor, with the guidance and support from the research team, designed three phases (six instructional sessions; each session lasted 45 min) in this course. The first four sessions (Phase I and Phase II) introduced the basic concepts of programming, including binary conversion, sequence, selection, and loops; the last two sessions (Phase III) introduced two advanced algorithms (i.e., sorting and searching). The design of the instruction sessions referred to the computer literacy development programs (CS Unplugged, 2020) and the book titled Computer Science Unplugged: Realizing Computing through Games and Puzzles (Bell et al., 2012). The instructor modified the instructional content and procedures to adapt local learners’ programming capacities. For instance, instead of sorting network, the instructor introduced the sorting algorithm with bubble sort activities, and also controlled the activities duration within 20 min according to the time limitation of the class. The content are required to be taught with Python in China’s high school according to the Information Technology Curricula for China’s high schools (MOE, 2020). During the instruction and learning processes, in the IDL class, learners received the instructor-directed lecturing with oral presentations to learn programming concepts and algorithms (see Fig. 2a). In the UPP class, the unplugged programming activities were intersected with the instructor’s lecturing; learners experienced 20-min unplugged programming activities in each session. For example, in the bubble sorting activity, learners held different paper cards, stood in a row randomly, and swapped with peers to make a correct sorting (see Fig. 2b).

### Data collection and analysis approaches

This research collected and analyzed data in four ways. First, we conducted pre- and post-test of learners’ computer programming knowledge and skills. The knowledge test included 12 questions, comprised of 10 multiple-choice questions on the programming concepts and 2 fill-in-blank questions related to the programming algorithms. Adapted from Computational Thinking Scale (CTS), learners’ computer programming skills were tested about the dimensions of creativity, algorithmic thinking, cooperativity, critical thinking, and problem solving. The CTS survey contained 5 dimensions and 29 measurement indicators (Korkmaz et al., 2017). Independent *t* test analysis was applied to compare the post-test of CT skills between two instructional modes.

Second, we recorded videos of two classes (without audios) to capture learners’ behaviors. We deliberately chose the last two courses classes (i.e., Phase III: the algorithm learning) as the video data for the current research (45 min/class; a total of 180 min). The reason that we chose the last two classes to collect behavior data was twofold. First, those two classes focused on two advanced algorithms that could better demonstrate programming capacities. Second, learners in the experimental class became more familiar with the procedures of the unplugged programming activities, such that they were more engaged in those two sessions as informal observation indicated. Video analysis was used to code learners’ in-class behaviors emerged during learning and instruction processes (Kersting, 2008). Video analysis followed an iterative coding process based on a previously validated coding framework (see D. Sun et al., 2021a, b). Two coders first separately watched the video recordings and wrote descriptive notes in excel files to identify initial codes of learners’ behaviors. Then, two coders had multiple meetings to discuss behaviors with conflicting codes and double checked the codes to achieve an agreement of the final coding framework (see Table 1). Finally, two coders independently coded the data again in a chronological order based on the coding framework, marked learner behaviors every 5 s, and cross-checked each other’s coding results. Two coders reached an inter-rater reliability with the Cohen’s Kappa of 0.801.

**Table 1:** Coding framework for classroom behaviors

Code	Description
Listening to Instructor (LtI)	Learners listened to the instructor during the class
Discussing with Peer (DwP)	Learners discussed with their partners during the class, including their discussions during the unplugged programming activities
Asking Questions (AsQ)	Learners asked questions to the instructor
Answering Question (AnQ)	Learners answered questions proposed by the instructor
Taking Notes (TN)	Learners took notes during the class
Irrelevant Behavior (IB)	Learners chatted, played or had other irrelevant behaviors

Furthermore, based on the video coding results, the lag-sequential analysis (LsA) was used to analyze learners’ behavioral patterns (Faraone & Dorfman, 1987), including the transitional frequencies between two behaviors and the visualized network representations in two instructional modes. There are five LsA measures, including (1) transitional frequencies (how often a particular transition occurred for a specified sequential interval); (2) expected transitional frequency (the expected number of times a transition would occur under the null hypothesis of independence or no relation between the codes); (3) transitional probabilities (the likelihood of occurrence of event B given that event A occurs); (4) adjusted residuals z scores (the statistical significance of particular transitions); (5) Yule’s *Q* (standardized measure ranging from − 1 to + 1 denoting strength of association) (Chen et al., 2017). Yule’s *Q* was finally adopted to represent the strength of transitional association, because it controls for base numbers of contributions and is descriptively useful (with a range from − 1 to + 1 and zero indicating no association). Moreover, using a previous network visualization method (Chen et al., 2017), this research presented LsA results in visualized networks, where the node size represented frequency of behavior code, the edge width represented transitional Yule’s *Q* value, and the transitional direction should be read from the node with the same color to the other node.

Regarding the differences of attitudes, pre- and post-surveys were conducted at the beginning and the end of the classes. The survey was adapted from the Georgia Computes project (Bruckman et al., 2009) and the Computing Attitudes Survey, which were validated from previous research (Dorn & Tew, 2015; Tew et al., 2012). The survey included five 5-point Likert scale questions ranging from 1 (strongly disagree) to 5 (strongly agree), as well as short open-ended questions (see Appendix A). Independent *t* test analysis and descriptive analysis were used to reveal the differences of learners’ confidence, enjoyment, and future interest between two instructional modes. Finally, we invited learners to a semi-structured interview at the end of the class. The interview focused on learners’ recall of the knowledge they learned from the class, the most difficult or easiest part of the class, as well as their self-perceptions and future plan on computer programming (see Appendix B). Thematic analysis was used to analyze the interview data (Cohen et al., 2013). A six-step sequence was used to identify themes: (1) formatting the text data, (2) coding the data separately by two coders, (3) recording specific coded segments of data, (4) comparing segments with same codes, (5) integrating the codes, and (6) double check the final coded themes.

## Results

### Computer programming knowledge and skills

We present the results of learners’ computer programming knowledge and skills on two dimensions, namely, the post-test scores and the score differences under two modalities (see Table 2). Regarding the pre-test programming knowledge at the outset of the research, no statistically significant (*t* (61) = 0.99, *p* = 0.32) was found between two instructional modes (IDL: *M* = 55.51, SD = 10.91; UPP: *M* = 58.28, SD = 11.11). After the intervention, learners in the IDL class had an average score of 68.70 (SD = 24.14), and learners in the UPP class gained an average score of 83.78 (SD = 10.33). *T* test indicated a statistically significant difference between two instructional modes (*t *(61) = − 3.20, *p* = 0.003) (see Table 2). Regarding the differences of knowledge score before and after the intervention, a significant difference (*t *(61) = − 2.46, *p* = 0.018) was found between two modes (IDL: *M* = 13.19, SD = 24.74; UPP: *M* = 25.50, SD = 12.94). These result indicated that learners in UPP class achieved significantly higher improvement on the knowledge assessment than peers in IDL class after the intervention. Moreover, regarding the scores of the CT skills, there were no significant differences between the IDL class (*M* = 3.94, SD = 0.88) and the UPP class (*M* = 3.92, SD = 0.94) (*t* (61) = − 0.23, *p* = 0.82) before the intervention. After the intervention, the independent *t* test results of post-test of learners’ CT skills indicated no statistically significant difference between two instructional modes (*t* (62) = − 0.26, *p* = 0.253), but the UPP class performed better than the IDL class overall (IDL: *M* = 4.07, SD = 0.45; UPP: *M* = 4.21, SD = 0.53). One significant difference was found on the sub item of *cooperativity* (*t* (62) = − 2.11, *p* = 0.042): the UPP class outperformed the IDL class (IDL: *M* = 3.75, SD = 0.62; UPP: *M* = 4.09, SD = 0.66). Regarding the differences of programming skill score, no significant difference (*t* (61) = − 1.30, *p* = 0.198) was found between two modes (IDL: *M* = 0.15, SD = 0.54; UPP: *M* = 0.38, SD = 0.86). Overall, compared to the instructor-directed lecturing class, the unplugged programming class had a better improvement on the programming knowledge and skills after the intervention.

**Table 2:** Independent *t* test of post-test of computer knowledge and skills in two instructional modes

	Dimensions	Modes	*M*	SD	*t*	*p*
Computer programming knowledge	Post-test scores	IDL	68.70	24.14	− 3.20^**^	0.003
		UPP	83.78	10.33		
	Differences (Post-test–pre-test)	IDL	13.19	24.74	− 2.46^*^	0.018
		UPP	25.50	12.94		
Computer programming skills	Post-test scores	IDL	4.07	0.45	− 0.26	0.253
		UPP	4.21	0.53		
	Differences (Post-test–pre-test)	IDL	0.15	0.54	− 1.30	0.198
		UPP	0.38	0.86		

### In-class behavioral patterns

**Fig. 3:**
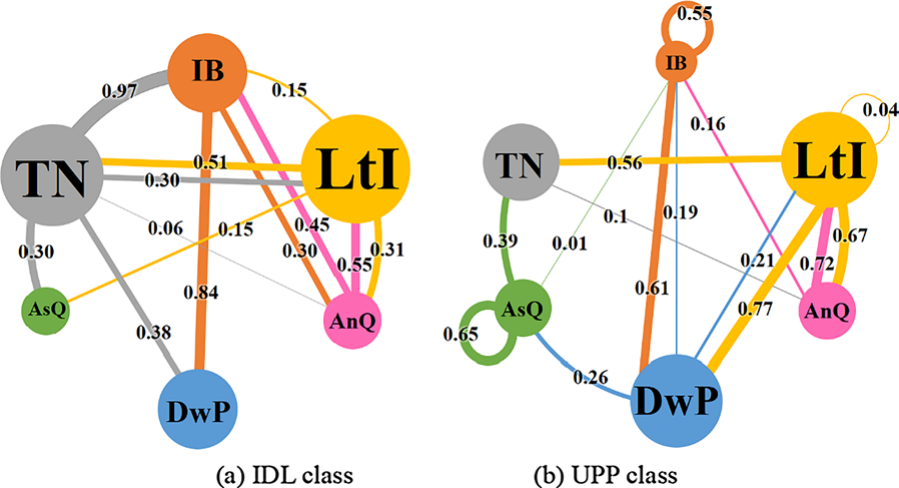
Transitional network representation in learners’ behavior from two instructional modes. A node represents a behavior code, the node size represented the frequency of the code, the width represented the transitional value, a Yule’s *Q* value, and the direction should be read from the node with the same color of the line to the node with a different color

Learners’ behavioral patterns showed similarities and discrepancies between two instructional modes. First, two classes had the most frequent behavior of listening to instructor (LtI), followed by either the behavior of discussing with peer (DwP) or taking notes (TN). In the IDL class, the most frequent behaviors were listening to instructor (LtI; frequency = 983), taking notes (TN; frequency = 831), and discussing with peer (DwP; frequency = 653). In comparison, learners in IDL class had much more irrelevant behaviors (IB; frequency = 441) than the UPP class (IB; frequency = 187), such as chatting or playing (see Fig. 3a). The most frequent behaviors of UPP class were listening to instructor (LtI; frequency = 757), discussing with peer (DwP; frequency = 736), and taking notes (TN; frequency = 509) (see Fig. 3b). Second, in the IDL class, the strongest association was IB → DwP (Yule’s *Q* = 0.84), followed by AsQ → LtI (Yule’s *Q* = 0.60) and AnQ → LtI (Yule’s *Q* = 0.55) (see Table 3). The results indicated that learners in the IDL class had most frequent behavior in irrelevant things and then transferred to discussing with partner and listening to the instructor. In the UPP class, the strongest association was LtI → DwP (Yule’s *Q* = 0.77), followed by AnQ → LtI (Yule’s *Q* = 0.72) and LtI → AnQ (Yule’s Q = 0.67). The results revealed that learners in the UPP class spent most of the time on listening to the instructor and then discussing with their partners. Taken together, the UPP class appeared to be more engaged (more behaviors in LtI and DwP, less behaviors in IB) during the instructional process, and the IDL class seemed to be more concentrated on listening to instructor (LtI) and taking notes (TN), while they were much easier to be distracted by irrelevant things (IB) during the class.

**Table 3:** LsA transition frequency of classroom behaviors of learners in two instructional modes

IDL		UPP	
Transition	Yule’s *Q*	Transition	Yule’s *Q*
IB → DwP	0.84	LtI → DwP	0.77
AsQ → LtI	0.60	AnQ → LtI	0.72
AnQ → LtI	0.55	LtI → AnQ	0.67
DwP → IB	0.54	AsQ → AsQ	0.65
LtI → TN	0.51	IB → DwP	0.61

Transitions with the top five Yule’s *Q* scores were presented

### Attitudinal findings

**Fig. 4:**
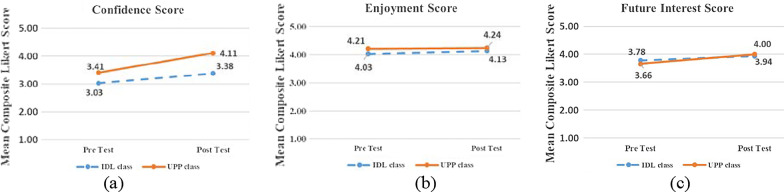
Scores of learners’ confidence (**a**), enjoyment (**b**) and future interest (**c**) in two instructional modes

We examined pre-test score, learning gains, and post-test score for both classes. First, learners in two modes had no significant difference in the pre-test of three dimension (confidence: *p* = 0.145; enjoyment: *p* = 0.491; future interests: *p* = 0.872). Second, learners in both classes experienced an improvement in three dimensions (see Table 4), including an increase of confidence: IDL (0.35), UPP (0.70); increase of enjoyment: IDL (0.10), UPP (0.03), increase of future interests: IDL (0.16), UPP (0.34). Regarding the differences before and after the intervention, no significant difference (*t* (61) = − 1.43, *p* = 0.156) was found on the confidence (IDL: *M* = 0.35, SD = 0.81; UPP: *M* = 0.70, SD = 0.44). No significant difference (*t *(61) = 0.38, *p* = 0.703) was found on the enjoyment (IDL: *M* = 0.10, SD = 0.64; UPP: *M* = 0.03, SD = 0.59). In addition, no significant difference (*t *(61) =  − 0.61, *p* = 0.547) was found on the future interest (IDL: *M* = 0.16, SD = 0.19; UPP: *M* = 0.34, SD = 0.38). Third, a significant difference was found in post-test score of confidence (*t *(61) =  − 1.47, *p* = 0.010). Learners in the UPP class (*M* = 4.11; SD = 1.03) were more confident than learners in the IDL class (*M* = 3.38; SD = 1.05) (see Fig. 4a). Although UPP class (*M* = 4.24; SD = 0.97) had a better enjoyment score than IDL class (*M* = 4.13; SD = 1.15), there was no statistically significant differences between two instructional modes (*t *(61) = − 0.57, *p* = 0.492) (see Fig. 4b). There was also no significant difference in the aspect of future interests (*t *(61) = − 0.94, *p* = 0.324), but learners in the UPP (*M* = 4.00; SD = 1.03) had a higher score than learners in IDL class (*M* = 3.94; SD = 1.03) (see Fig. 4c). Overall, UPP class had an overall more positive attitude towards computer programming than IDL class.

**Table 4:** Independent *t* test of post-test of attitudinal findings under two instructional modes

	Dimensions	Modes	*M*	SD	*t*	*p*
Confidence	Post-test scores	IDL	3.38	1.05	− 1.47^*^	0.010
		UPP	4.11	1.03		
	Differences	IDL	0.35	0.81	− 1.43	0.156
		UPP	0.70	0.44		
Enjoyment	Post-test scores	IDL	4.13	1.15	− 0.57	0.492
		UPP	4.24	0.97		
	Differences	IDL	0.10	0.64	0.38	0.703
		UPP	0.03	0.59		
Future interest	Post-test scores	IDL	3.94	1.03	− 0.94	0.324
		UPP	4.00	1.03		
	Differences	IDL	0.16	0.19	− 0.61	0.547
		UPP	0.34	0.38

### Qualitative analysis of interview data

There were three themes emerged in the thematic analysis of learners’ interview data, namely, the recall of programming knowledge, feeling of learning experiences, and attitudes towards programming (see Table 5). The first theme revealed differences of acquisitions of programming knowledge and skills between two instructional modes. 18 out of 31 learners in IDL mentioned that it was hard for them to recall the contents of the class, and 4 learners expressed that they were easily confused by the divergent contents of each class. Huang said, “I thought it was ok, but the technical terms and calculation methods of computers may be too difficult for me, and I was often confused by different rules.” Ye mentioned, “I have some impressions of what I have learned in this course, but I didn't master the rules and methods very well from the class, because I don't have a chance to consolidate them after class.” As for UPP, 20 out of 32 learners mentioned that they could remember most of the content of each class, and they thought unplugged activities improved their higher-order thinking ability. For example, Zhang said “I could recall most of the class content, such as sorting, searching…. What impressed me most was to the activity of moving the black and white block to find the correct sequence… activities like these made me remember the algorithm better than just sitting and listening to the instructor.” Only 3 learners in UPP class thought it was difficult for them to master the instructional content through unplugged activities. Liu said, “I was attracted to the unplugged activities during the class, but sometime I found it hard to recall the corresponding programming concepts”. Overall, learners in the UPP class had a better understanding of programming content and concepts, compared with IDL class.

**Table 5:** Themes extracted from semi-interviews in two instructional modes

Themes and sub-themes	IDL *N*	UPP *N*
Recall of programming knowledge		
· Difficulties of recalling course contents	18	3
· Confusion about course contents	4	6
· Recall of most of the course contents	8	20
Feeling of learning experiences		
· A low level of participation	22	3
· A lack of opportunity to conduct programming practices	8	0
· An interactive and interesting learning experience	2	26
Attitudes towards programming		
· Concerns about the difficult level of the algorithms	15	5
· Positive attitudes towards computer programming	9	27
· Confidence about other STEM subjects, such as mathematics	4	10

The second theme revealed the difference of learners’ feeling of learning experience between two instructional modes. 22 out of 31 learners in IDL class referred to a low level of participation in the class, as Yang said: “There is nothing special about this course, the learning experience was poor, since we did not have chance to practice the algorithm by ourselves or through computer.” Two learners in IDL thought the class was interesting, Huang said “I was interested in the class because I was attracted to different algorithms like bubble sort”. On the contrary, much more learners (*N* = 26) in UPP class described the unplugged programming as an interactive and interesting learning experience. Su mentioned: “…we had a lot of opportunities to join in the programming activities during class, which promoted our concentration and engagement.” But three learners in UPP class mentioned participation issues during unplugged activities, as Chen said “…sometimes it was difficult for me to get the idea quickly for the unplugged activities, so I had to follow others in my group.” Overall, UPP class appeared to be more interactive and engaging compared to the IDL class.

The third theme of perceptions discussed learners’ attitudes towards computer programming between two instructional modes. Fifteen learners in IDL expressed interests in programming, but they appeared to be more concerned about the difficulty level of the algorithms considering their mathematical abilities. Sun said, “I thought this course was fine, but the course seemed to have something to do with the mathematics ability. I could use some basic knowledge to solve problems… but when it got harder and deeper, I was not able to handle it.” Nine learners in IDL thought the class improved their attitudes towards programming and four learners mentioned the programming class was beneficial to other subjects which required computational thinking ability. Most of the learners (*N* = 27) in the UPP class mentioned that learning through unplugged programming activities could promote their learning attitudes and 10 of them mentioned the programming class could improve their performances in other subjects, especially mathematics, which was consistent with previous research (e.g., Century et al., 2020). For example, Wang responded, “Some knowledge within the unplugged programming activities were connected with our mathematics course, such as sequence… I think it is quite suitable for me.” Huang said, “I might not be majoring in computer science in the future, but I think the profession I choose in the future will involve computer science knowledge, so I think it was worth learning.” There were few learners (*N* = 5) expressed their concern on the difficulty of the algorithms. Taken together, UPP seemed to offer the opportunity to improve learners’ attitudes and alleviate their concerns for computer programming. Overall, interview data showed that learners in UPP were more confident in mastering the computer knowledge and skills, more engaged during the classes, and had more positive feelings towards programming.

## Discussion

As one area of STEM education, computer programming focuses on transforming the instructor-directed lecturing to the learner-centered instructions (such as unplugged, game-based programming) to foster learners’ computational thinking skills, learning motivations and interests, as well as programming engagement (Koretsky et al., 2018; Looi et al., 2018; Tekkumru-Kisa & Stein, 2017). This research used a quasi-experimental design to apply two instructional modes, namely, the instructor-directed lecturing and the learner-centered unplugged programming, to foster computer programming in China’s secondary education. Furthermore, this research compared the effects of novice learners’ programming between those two instructional modes, including knowledge gains, in-class behaviors, and attitudinal changes. The research results revealed discrepancies between two instructional modes. First, learners in the unplugged programming class achieved significantly higher scores on the knowledge tests, compared to learners in the traditional lecturing class. The results echoed with Grover et al. (2019)’s research that found unplugged programming activities deepened novice learners’ understanding of programming concepts. Consistent with previous research results (Hsu & Liang, 2021), compared to the traditional lecturing class, learners in the unplugged programming class achieved higher scores of computational thinking skills, particularly on the cooperativity dimension. The results together indicated that learners benefited from unplugged programming to improve knowledge gains as well as computational thinking skills. Next, discrepancies of in-class behaviors showed that the typical behaviors in unplugged programming class were listening to the instructor’s lectures and discussing with peers during unplugged programing activities, while learners in the instructor-directed lecturing class had frequent behaviors of listening to the instructor, taking notes, and irrelevant behaviors. Consistent with previous research (e.g., Ballard & Haroldson, 2021; Huang & Looi, 2020), unplugged programming activities reduced irrelevant in-class behaviors, promoted peer discussions, and facilitated students’ problem-solving process. Results of attitudes showed a significant difference on the confidence dimension between two instructional modes: learners in the unplugged programming activities self-reported a higher level of confidence than learners in the traditional class. Qualitative analysis of interview data also confirmed those quantitative results. Echoing with previous studies (Brackmann et al., 2017; del Olmo-Muñoz et al., 2020; Price & Barnes, 2015), this research revealed that the learner-centered unplugged programming had potential to improve learners’ programming knowledge, behaviors, and attitudes compared to the traditional instructor-directed lecturing mode.

Based on the results, this research proposes pedagogical and analytical implications for future instructional design and learning analytics of unplugged programming. First, on the pedagogical level, instructors should consider integrating unplugged programming activities in daily instructions for novice learners, with an aim to provide conceptual contextualization, material supports, and peer interaction opportunities (Alamer et al., 2015). Our results showed that, compared to the traditional lecturing class, learners in the unplugged programming class seemed to be more attracted to the instructional content and more concentrated on learning with less irrelevant behaviors. Learners in unplugged programming class had more behaviors of peer discussions, questioning and answering, which was critical for improving the cognitive quality during programming (Lu et al., 2017). Our results also revealed that learners in the instructor-directed lecturing mode mentioned two main barriers which might lead to difficulties of knowledge acquisition: insufficient learning time and a lack of opportunity for programming practices. An integration of the unplugged programming activities could be beneficial to address those challenges, since those hands-on activities bring more opportunities for learners to engage in actual programming practices. In this way, instructors can deliver programming knowledge and skills through pragmatic practices, which, in turn, would facilitate learners’ questioning, thinking, and reflection of programming (Huang & Looi, 2020). Learner agency can be also promoted through unplugged programming practices to increase learners’ intentionality for and their action of taking learning initiations (Bandura, 2001). Overall, the unplugged programming activity is suggested for instructors to integrate in daily instructions to increase peer interaction and collaboration opportunities, to maintain learners’ motivation and interest of programming, and to increase the overall learning quality of programming.

On the analytical level, there has been a trend currently to apply the mixed method (e.g., clickstream analysis, behavior sequential analysis, statistical analysis) to conduct the process-oriented analytics of computer programming (e.g., D. Pereira et al., 2020; Sun et al., 2021a, b; Wu et al., 2019). Although final performance is usually the main focus in education (Zhong et al., 2016), the process-oriented perspective highlights the importance of using multiple learning analytics to evaluate programming and emphasizes the essence of promoting learners’ programming quality through pragmatical practices. As the analytical framework indicates (see Fig. 1), the process-oriented and summative assessment complements each other to provide a holistic insight into learners’ programming processes and performances; with the support of an integrative assessment, researchers can better understand the programming phenomenon and underlying factors that may influence the programming process. Specifically, findings from pre- and post-tests of computer programming knowledge and skills provide us with a general description of learners’ improvement before and after the intervention; network representations reveal a process-oriented behavioral transition during the instructional process; and qualitative interview analytics discover learners’ in-depth perceptions of the programming after the intervention. Moreover, mixed methods provide a broader view of the computer programming phenomenon under investigation, clarify and answer research questions from varied perspectives, enhance the validity of the research findings and increase the capacity to cross-check one data set against another (Grbich, 2013). However, due to the technical restriction, we were not able to capture learners’ in-class behaviors and their communicative discourses synchronously; such that we were not able to conduct a more integrated microanalysis of the moment-to-moment details of how learners coordinate their communications, behaviors, and movements during the programming processes (Stahl, 2009). Multimodal learning analytics could be integrated into future research to synchronize audio discourse data, video recording data, facial expressions and eye tracking movements to better reveal the programming learning patterns (e.g., Chevalier et al., 2020; Sun & Hsu, 2019; Zatarain Cabada et al., 2018). Overall, complementing each other, the summative and process-oriented instructional design and analysis are promoted based on the empirical results, to provide a more holistic, multilevel, multidimensional analysis of the unplugged programming processes.

Programming education focuses on cultivating learners’ higher order thinking abilities (e.g., computational thinking and logical thinking), which are fundamental skills that modern learners should possess (Stehle & Peters-Burton, 2019). The unplugged programming strategy can be easily integrated into various types of computer programing classes, which is beneficial to improve learners’ knowledge gains, classroom learning behaviors, and positive attitudes and motivations towards programming, as this research demonstrates. Unlike learning professional programming languages (e.g., C, Java, Python), the instructional mode of unplugged programming makes programming knowledge accessible to novice learners with different backgrounds, serves as the basis for learners to make further explorations, and enhances learners’ higher order cognitive abilities and computer thinking capacities (Bell & Vahrenhold, 2018; Thies & Vahrenhold, 2013). As an alternative to formal education of computer programming, unplugged programming has been designed and implemented in China and other countries all over the world, proved to be a flexible, feasible form for a wide range of learners to learn computer programming (Huang & Looi, 2020). In formal and informal learning, instructors can integrate various unplugged programming strategies into in-class instructions to promote learners’ learning efficiency and further expand the coverage of programming education (Looi et al., 2018). Since the instructor-directed lecturing is the main instructional mode of computer programming in formal educational context in China and many other countries (Panwong & Kemavuthanon, 2014; Wu & Wang, 2017), instructors might found it hard to integrate unplugged activities in their daily classes. Moreover, instructors might meet other difficulties during implementations of unplugged programming activities, including design of unplugged activities to illustrate computer knowledge and content, suit for learners with divergent level of prior knowledge and skills, and class time allocation of unplugged activities and other instructional lectures (Taub et al., 2009; Torres-Torres et al., 2019). To deal with these challenges, the instructor should carefully identify relationships between unplugged programming activities and central programming concepts and algorithms when designing and preparing lesson plans, course materials, and programming activities (Brackmann et al., 2017; Taub et al., 2012). In addition, this research suggests that the instructor should take into consideration learners’ pre-existing knowledge and skill levels when implementing unplugged programming activities to achieve a learner-centered programming practices. Taken together, since the effect has been validated by educational research, unplugged programming, as a computer-science-for-all strategy in formal education, has potential to bring practical and pragmatic benefits into formal programming education.

## Conclusions, limitations, and future directions

Computer science education plays an important role in STEM education to foster the learner-centered learning. As a feasible and easy-to-use instructional activity in computer science education, unplugged programming is encouraged to be integrated in formal education to transform education from the instructor-directed lecturing to the learner-centered learning with an aim to increase learners’ learning interests and motivations (Alamer et al., 2015; Looi et al., 2018; Sun et al., 2021a, b). This quasi-experimental research compared learners’ programming knowledge, behaviors, and attitudes under two instructional modes, namely, the instructor-directed lecturing and the learner-centered unplugged programming, in China’s secondary education. The results revealed critical discrepancies between two instructional modes on learners’ knowledge gains, classroom learning behaviors, and changes of attitudes towards programming.

A major limitation of this research is the relatively short period of time of learning in the research. This research chose the last two sessions as the data source for process-oriented behavioral analysis, which might cause selection bias to some extent, since learners showed the highest level of engagement in this period. Therefore, future research should expand the research duration, such as collecting data from the whole instruction and learning process. Another limitation is the possibility of Hawthorne effect due to the instructor’s enthusiasm and attention for the treatment class (Chia & Lim, 2020). To eliminate the bias, future research should extend the sample size to further validate the proposed implications. Furthermore, as the proposed analytical framework suggests, this research investigated the process and performance data from behavioral, summative, and attitudinal perspectives. Moreover, following the proposed analytical framework, multimodal learning analytics (MLA) has potential to guide a better process-oriented analysis for discovering frequent patterns of behaviors, gestures, emotions, and communications during instruction and learning processes (Ochoa, 2017). In computer programming research, MLA can collect and analyze multimodal data (e.g., audio/video recording data, click-stream recording data, facial expressions, movement and gesture, and eye tracking, etc.) to reveal learners’ coordination of behavioral, cognitive, metacognitive, and social activities of programming (e.g., Wiltshire et al., 2019).

Overall, since the intrinsic value of programming centers on its process, relevant research and practice should integrate instructor-directed lecturing with learner-centered unplugged programming and take a process-oriented perspective to investigate, advance, and assess learners’ programming. This research takes a step forward to conduct a holistic analysis of learners’ performances, processes, and attitudes in computer programming education in China’s formal secondary education. Based on the empirical research results, unplugged programming has shown its flexibility and practicability for a wide range of learners to improve their programming knowledge gains, behaviors, and positive attitudes. Overall, it is highly suggested that computer programming education should integrate unplugged programming with traditional lectures in formal education to promote learners’ programming knowledge, programming engagement, and positive attitudes towards programming.

### Supplementary Information

**Additional file 1.** Behavior data for the IDL mode.

**Additional file 2.** Behavior data for the UPP mode.

## Data Availability

The data was available upon request from the corresponding authors.

## Appendix A

### Attitudinal survey

1. I will be/am good at programming.
2. I will be doing well/did well in this course.
3. I like programming.
4. I am excited about this course/ I was excited about this course.
5. I might take more programming courses in the future/ I plan to take more programming courses in the next semester.

## Appendix B

### Interview

1. Do you like computer programming and why?
2. What do you think of this course so far?
3. Can you recall what you learned in this course? What is the most impressive part of the course?
4. What do you think is the easiest or hardest part of this course?
5. What abilities have you improved after this course?
6. What is your future plan on learning of computer programming?
